# Permeabilization of tumor margins in brain macrometastases using a type-1 receptor selective tumor necrosis factor mutein

**DOI:** 10.1093/noajnl/vdag239

**Published:** 2026-09-08

**Authors:** Vanessa A Johanssen, Claire Bristow, Sandra J Campbell, Daniel C Anthony, Mark R Middleton, Nicola R Sibson

**Affiliations:** Department of Oncology, University of Oxford, Oxford, UK; Department of Oncology, University of Oxford, Oxford, UK; Department of Oncology, University of Oxford, Oxford, UK; Department of Pharmacology, University of Oxford, Oxford, UK; Department of Oncology, University of Oxford, Oxford, UK; Department of Oncology, University of Oxford, Oxford, UK

**Keywords:** brain metastasis, mutein, permeabilization, tumor margins, tumor necrosis factor

## Abstract

**Background:**

Brain metastasis remains a major clinical challenge. The blood-brain barrier, which persists in the margins even in late-stage macrometastases, is a major impediment to effective drug delivery, surgery, or radiotherapy. The aim of this study was to determine whether a mutein of human tumor necrosis factor (TNF) that is selective for the TNFR1 receptor could increase permeabilization in the margins of brain metastases, enabling better delineation and more complete treatment.

**Methods:**

Intracerebral xenograft mouse models of breast cancer (MDA231Br-GFP), melanoma (H1_DL2), and lung cancer (SEBTA) brain metastasis were used to test the efficacy of the TNF mutein (mutTNF) in permeabilizing the margins of well-established macrometastases. Changes in tumor permeabilization were assessed in vivo by gadolinium-enhanced magnetic resonance imaging (MRI) and ex vivo by IgG immunohistochemistry.

**Results:**

Tumor margin permeabilization was increased following mutTNF treatment in all 3 models. The MDA231Br-GFP model showed a significant increase in the extent of both gadolinium enhancement (*P* < 0.01) and IgG extravasation (*P* < 0.05), whilst significant effects were detected by MRI alone in the SEBTA (*P* < 0.01) and H1_DL2 (*P* < 0.05) models. Although a strong trend toward increased IgG extravasation was found in the SEBTA model (*P* = 0.076). Consistent with these findings, the models showed differing degrees of both infiltration and vascular TNFR1 expression at the margins (MDA231Br-GFP > SEBTA > H1_DL2), suggesting a greater sensitivity to mutTNF with increasing marginal invasiveness.

**Conclusions:**

This work demonstrates the potential of TNFR1-selective mutTNF as an adjuvant strategy to improve tumor margin delineation and enhance therapeutic access in brain metastases.

Key Points

- TNFR1-selective TNF mutein induces permeabilization at the margins of brain macrometastases.
- The effect of TNF mutein is more evident in highly proliferative and invasive brain metastases.

Importance of the Study

Brain metastasis remains a major clinical challenge. By the time brain metastases can be diagnosed with traditional imaging methods, they are by default in the later stages of development. Although the blood-brain barrier (BBB) may be permeable in the core area of the tumor at this stage, it remains intact at the tumor margins. The BBB excludes ∼98% of small molecule therapies, most antibodies, and common imaging contrast agents. Consequently, the margins are frequently incompletely treated either by systemic therapies or by surgery and/or radiotherapy. It is in these regions that recurrence often occurs. In this study, we have demonstrated that a TNFR1-selective mutant human TNF protein (mutTNF) enhances permeabilization at the margins of later-stage brain metastases. Moreover, as the invasiveness of the tumors increases, so does the efficacy of mutTNF in increasing margin permeabilization. Consequently, this approach may enable more complete treatment of brain metastases, reducing later recurrence.

Metastatic spread to the brain is a significant clinical problem,^1^ with breast, melanoma, and lung primary cancers showing the greatest propensity toward brain progression. Clinically, brain metastases are diagnosed only when they are sufficiently large to be detected with magnetic resonance imaging (MRI), by which time prognosis is poor. Although advances in some therapeutic modalities have improved outcomes for particular subsets of patients, brain metastasis remains a marker for poor outcomes and high treatment costs.^1^ As treatment of primary and systemic metastatic disease improves, more effective treatment of brain metastases is increasingly urgent. A significant hurdle to the treatment of brain metastases is the blood-brain barrier (BBB). The BBB is intact during the early *micro*metastatic stages of tumor development and becomes only heterogeneously permeable in established *macro*metastases, remaining largely impermeant in the invasive margins.^2^ Consequently, the presence of an intact BBB excludes systemic therapies from both *micro*metastatic sites and from the highly proliferative margins of *macro*metastases.

As systemic metastases are treated more effectively, it is critical that we find ways to overcome BBB function and enable more effective treatment of brain metastases.^2^^,^^3^ A number of strategies have been developed to overcome the restrictions imposed by an intact BBB,^4-9^ but these all suffer 1 or more limitations, including transient windows of permeability (<1 h), size-restricted access (<1 kDa), lack of targeting to tumor sites, and dose-limiting toxicity.

We have previously shown that intravenous injection of tumor necrosis factor (TNF) selectively permeabilizes the BBB at sites of *micro*metastases in the brain when the BBB otherwise remains intact.^10^ We have further shown that this permeabilization is mediated specifically by the TNF type 1 receptor (TNFR1), which is selectively upregulated on the vascular endothelium closely associated with brain metastases, but not on normal brain vasculature. We subsequently showed that a TNFR1-selective mutein of TNF (mutTNF) similarly induces BBB permeabilization selectively at sites of brain *micro*metastases.^11^ As a result, it is possible to induce selective permeabilization at the *micro*metastatic stage, when these tumors are undetectable by conventional diagnostic imaging methods. This approach offers several advantages, including a prolonged permeabilization window (24-72 h), entry of agents across a clinically relevant size range (0.5-150 kDa), selective permeabilization at metastatic sites, and the ability to target all brain metastases with no prior knowledge of their location.

Whilst the above represents a significant potential step forward in the earlier and more effective treatment of brain *micro*metastases, there remains the issue of more established brain tumors that are often incompletely treated by conventional methods. A first local recurrence is common after resection or radiotherapy for brain metastasis. Even following optimal surgical resection, local recurrence rates at the original metastatic site varies from 21%-46%.^12^^,^^13^ Although postoperative stereotactic radiotherapy can improve the outlook, significant numbers of patients will still suffer recurrence.^12^ Following standalone stereotactic radiosurgery (SRS), recurrence rates are as high as 73%-76%, falling to 27%-46% on combination with whole-brain radiotherapy.^14^ Although not as typically diffuse in morphology as glioblastoma, recent studies have revealed the infiltrative nature of many brain metastases,^15-17^ albeit in a more heterogeneous fashion. It is these infiltrative marginal regions that can be left untreated by conventional methods, such as SRS, owing to uncertainty in the delineation of tumor boundaries and a conservative approach to preserve uninvolved brain tissue. As a result of an intact BBB in these marginal areas, they are not detected by diagnostic imaging and evade systemic therapies that can only penetrate areas of the tumor in which the BBB is compromised.

We have previously shown that the peripheral regions of more established *macro*metastases in mouse brain and also human brain metastases show marked upregulation of inflammatory indices,^18^ including upregulation of TNFR1.^10^ Given this, we hypothesized that our TNFR1-targeted approach may enable permeabilization of the infiltrative margins of more advanced brain *macro*metastases. The primary aim of this study, therefore, was to determine whether administration of mutTNF would increase the extent of BBB permeabilization in established *macro*metastases, particularly in marginal areas. To this end, we assessed the extent of mut-TNF-induced BBB permeabilization across 3 different models of brain *macro*metastasis, from primary breast, lung, and melanoma cancers that exhibited differing degrees of infiltration and invasiveness.

## Methods

### Cell Lines

Three human-derived cell lines were used: (1) breast carcinoma MDA231Br-GFP cells (kind gift from Dr. P. Steeg, United States); (2) lung adenocarcinoma SEBTA-001 cells (kind gift from Prof. G. Pilkington, United Kingdom); and (3) melanoma H1_DL2 cells (kind gift from Prof. F. Thorsen, Norway). MDA231Br-GFP cells were cultured in Dulbecco’s modified Eagle’s medium (DMEM; Sigma Aldrich) containing 10% Fetal Bovine Serum (FBS; Labtech UK) and 1% L-glutamine (Life Technologies). SEBTA-001 cells were maintained in DMEM supplemented with 2% human serum (Sigma Aldrich). H1_DL2 cells were cultured in ALT-DMEM (DMEM supplemented with 10% FBS [Labtech, United Kingdom], 2% L-glutamine (Life Technologies), and 4 times the prescribed concentration of non-essential amino acids (Gibco, Thermo Fisher Scientific, United Kingdom)). Cells were tested for *Mycoplasma* before in vivo injection, using the MycoAlert Mycoplasma Detection Kit (Lonza). Cells were kept in a standard tissue culture incubator at 37 °C with 100% humidity and 5% CO_2_.

Short tandem repeat (STR) profiling has been provided for the H1_DL2 cells by Prof. F. Thorsen and for the SEBTA-001 cells by Prof. G. Pilkington. Short tandem repeat profiling for the MDA231Br-GFP cells has been performed by Eurofins Genomics UK Ltd; however, work was conducted from the original cell stock for all cell lines. Moreover, all experiments within a study were run from the same passage from this original stock to ensure reproducibility, as far as possible.

### Mouse Models of Brain Macrometastasis

All animal experiments were approved by the University of Oxford Clinical Medicine Ethics Review Committee and the UK Home Office (Animals [Scientific Procedures] Act 1986) and conducted in accordance with the University of Oxford Policy on the Use of Animals in Scientific Research, the ARRIVE Guidelines, and Guidelines for the Welfare and Use of Animals in Cancer Research. All mice were maintained in a specific pathogen-free environment and were acclimatized for a minimum of 7 days before any experimental procedures. Brain metastases were induced in female SCID mice via direct intracerebral injection of 5000 human breast cancer (MDA231Br-GFP; *n* = 16), lung cancer (SEBTA-001; *n* = 18), or melanoma (H1_DL2; *n* = 15) cells into the left striatum in 0.5 μL saline, as described previously.^19^ At day 21 (H1_DL2) or day 28 (MDA231Br-GFP; SEBTA-001), animals underwent contrast-enhanced MRI to assess the extent of natural BBB breakdown. Animals that did not meet the predefined tumor burden criterion at the initial imaging time point were re-assessed once at a later time point (2-5 days later H1_DL2; 1-3 days later SEBTA-001). No animal underwent more than 2 MRI assessment time points during the study.

### MRI Assessment of BBB Permeability

Mice underwent T_1_-weighted MRI both pre- and post-intravenous injection of gadolinium-DTPA (Gd-DTPA; 30 μL Omniscan; via tail vein cannula) to assess BBB breakdown, as described previously.^11^ Once metastatic foci were detectable by post-gadolinium T1-weighted MRI and the area of contrast enhancement encompassed ≥20% of the left striatal area (day 28 MDA231Br-GFP; days 28-31 SEBTA; days 21-26 H1_DL2), animals were randomly assigned using a coin flip simulator to treatment or control groups and injected intravenously with either mutTNF (150 µg/kg in 100 µL saline) or saline alone (100 µL). Pre- and post-Gd-DTPA T1-weighted MRI datasets were re-acquired 2 h after treatment to identify extent of BBB breakdown, as above. Following MRI, mice were perfusion-fixed for subsequent immunohistochemical analysis.

To assess the volume of BBB breakdown detectable by contrast-enhanced MRI, both raw post-Gd-DTPA images and difference images were analyzed. In the first instance, the volume of contrast enhancement was determined at metastatic sites on the raw post-Gd-DTPA T_1_-weighted images acquired both before treatment and 2 h after treatment. Image signal intensity was set to a consistent level, and areas of signal enhancement were segmented out and quantified. In the second case, the pre-Gd-DTPA images were subtracted from the post-Gd-DTPA T_1_-weighted images, for both the pre- and post-treatment datasets. The same analysis approach as above was subsequently applied, and the resulting volumes quantified. All datasets were analyzed blind to treatment group.

### Immunohistochemical Analysis of IgG Extravasation

At the end of each experiment, animals were transcardially perfusion-fixed under terminal anesthesia with 0.9% heparinized saline, followed by 50 mL of by PLP_light_ fixative (75 mM l-lysine monohydrochloride, 10 mM Na_2_HPO_4_, 2% w/v paraformaldehyde, 1 mM sodium metaperiodate in phosphate buffer, 0.025% w/v glutaraldehyde, pH 7.2). Brains were postfixed in PLP_light_, cryoprotected, embedded, and frozen in isopentane at −40 °C. Subsequently, 20 μm thick coronal sections were cut through the striatum, from *ca.* +1.42 mm to *ca.* −1.70 mm relative to bregma and mounted sequentially on glass slides in sets of 3.

Every third slide was stained immunohistochemically for IgG extravasation into the brain tissue as a marker of BBB permeabilization. Slides were quenched with 1% hydrogen peroxide in methanol and blocked with 0.5% blocking reagent (PerkinElmer, Waltham, Massachusetts, United States) diluted in 0.1 M TRIS hydrochloride and 0.15 sodium chloride (TNB blocking buffer), for 1 h at room temperature. Sections were incubated with primary biotinylated goat anti-mouse IgG antibody (1:1000; Vector Laboratories, California, United States) in TNB blocking buffer overnight at 4 °C. This step was followed by incubation with the avidin/biotin complex-HRP (VECTASTAIN Elite ABC Kit Standard, Vector Laboratories, California, United States). Peroxidase was detected using 3,3′-diaminobenzidine (DAB) and sections were counterstained with 0.5% cresyl violet. Tissue was dehydrated using increasing concentrations of ethanol, cleared in xylene, and mounted in DPX mountant (Fisher Scientific, United Kingdom).

To determine the extent of BBB breakdown post-treatment, immunostained sections were imaged with an Aperio ScanScope CS (Leica) slide scanner and analyzed using Aperio ImageScope v12 (Leica). An automated program was used to quantify all positive and negative pixels within a region of interest (ROI). First, an ROI was drawn around the tumor foci on each section, and the total number of pixels (positive and negative) therein was counted. Subsequently, the total number of positive pixels (brown IgG-positive stain) in each hemisphere was calculated. Since background staining varied between sections, the number of positive pixels in the contralateral hemisphere was subtracted from that of the tumor-bearing hemisphere using equivalent ROIs. The resulting number of pixels for IgG-positive staining was subsequently divided by the number of tumor pixels in the same section to normalize between animals and reduce variability owing to differences in tumor size. Three sections through the middle region of each tumor were analyzed per brain and averaged.

Animals were identified in a way that only the specific experiment they participated in could be determined. All datasets were analyzed blind to treatment group. A small number of animals were excluded from either the MRI and/or IHC analysis in the MDA231Br-GFP and SEBTA groups owing to poor imaging or immunohistochemistry (IHC) data quality. Consequently, final group numbers for all analyses were: MDA231BR-GFP—saline *n* = 7, mutTNF *n* = 8; SEBTA—saline *n* = 7, mutTNF *n* = 8; H1_DL2—saline *n* = 6, mutTNF *n* = 6.

### Immunohistochemical Analysis of TNFR1 Expression

Either 1 or 2 slides adjacent to those stained for IgG at the center of the tumor foci were stained for TNFR1 to assess the extent of upregulation. Slides were rehydrated in PBS, then placed into 1× sodium citrate for antigen retrieval. After two 5 min washes in PBS with 0.05% Tween (PBST) then PBS, slides were quenched with 1% hydrogen peroxide in methanol, washed in PBS for 2 × 5 min, then washed in PBST for 5 min. Endogenous biotin was then blocked using a streptavidin/Biotin blocking kit (Vector Laboratories, California, United States) following manufacturer’s protocol. Using the Invitrogen Image-it FX solution (Thermo Fisher Scientific, United Kingdom), signal enhancement was applied following the standard protocol. Slides were then blocked in TNB blocking buffer for 1 h at room temperature. Sections were incubated with Anti-TNF Receptor I primary antibody (1:50 Abcam, Cambridge, United Kingdom) in TNB blocking buffer overnight at 4 °C. After washing in PBS 3 × 5 min, slides were incubated in biotinylated Goat Anti-Rabbit IgG antibody (1:100 Vector Laboratories, California, United States) in TNB blocking buffer for 1 h 30 min at room temperature. After washing in PBS 3 × 5 min, streptavidin-HRP solution (1:200 TSA Biotin System, PerkinElmer, United States) in TNB blocking buffer was applied for 30 min, washed in PBS 3 × 5 min, then incubated in the Biotin Tyramide working solution (1:100 in amplification buffer; TSA Biotin System, PerkinElmer, United States) for 7 min. Once washed 3 × 5 min in PBS, the slides were incubated with the avidin/biotin complex-HRP at 1:100 (VECTASTAIN Elite ABC Kit Standard, Vector Laboratories, California, United States) in TNB blocking buffer. Peroxidase was detected using DAB, and sections were counterstained lightly with 0.5% cresyl violet. Tissue was dehydrated using increasing concentrations of ethanol, cleared in xylene, and mounted in DPX mountant (Fisher Scientific, United Kingdom). Sections were assessed qualitatively for vascular TNFR1 expression both within and surrounding the tumor foci.

### Statistical Analysis

For these studies, we have no previous data indicating a likely effect size. However, with 8 animals per group, based on a significance level of 0.05, the power to detect a 20% biologically relevant change is 99%; therefore, we aimed to have a minimum of 8 animals per group. Statistical analysis was performed using Prism (GraphPad Software Inc.). To assess differences in the volume of contrast enhancement on MR images post-treatment vs pretreatment in the same animals, a Wilcoxon matched-pairs test was used. To determine differences in the extent of IgG extravasation in mutTNF-treated animals vs control saline-treated animals, a Mann-Whitney *U*-test was used. All statistical tests were based on the hypothesis that the extent of BBB permeabilization would increase in mutTNF-treated animals compared either to pretreatment in the same animals or to control animals and, consequently, were one-tailed. To confirm that the tumor sizes were comparable between saline and mutTNF-treated groups a 2-tailed *t* test with a Welch correction was used. Significance was assumed at *P* < 0.05.

## Results

### Assessment of BBB Breakdown by Contrast-Enhanced MRI

To assess BBB permeabilization in vivo, contrast-enhanced MRI was used. Extravasation and accumulation of Gd-DTPA enable BBB breakdown to be detected in vivo as, hyperintense areas on T_1_-weighted MR images. Both raw post-Gd-DTPA T_1_-weighted and difference images indicated small regions of contrast enhancement in all animals prior to treatment with either saline (Figure S1A-C) or mutTNF (Figure S1D-F), indicating levels of natural BBB breakdown at this stage in tumor development. Assessment of the volume of MRI signal enhancement prior to treatment showed no significant difference between the control and treated groups for any of the models, from either the raw post-Gd-DTPA T_1_-weighted images or the difference images (Figure S1G-I). These findings indicate a comparable level of natural BBB breakdown between treatment groups.

When comparing pre- and post-treatment MRI data, both raw post-Gd-DTPA T_1_-weighted images and difference images, some variability between the models was evident. In the majority of saline-treated animals, the volumes of contrast enhancement showed either a decrease or negligible change post-treatment (Figure 1), with the decrease just reaching significance (*P* < 0.05) in the difference data from the MDA231Br-GFP group (Figure 1A). These findings indicate broadly similar regions of contrast enhancement pre- and post-treatment, which can yield an apparent decrease in the difference images, depending on the signal-to-noise variability between scans.

**Figure 1.**
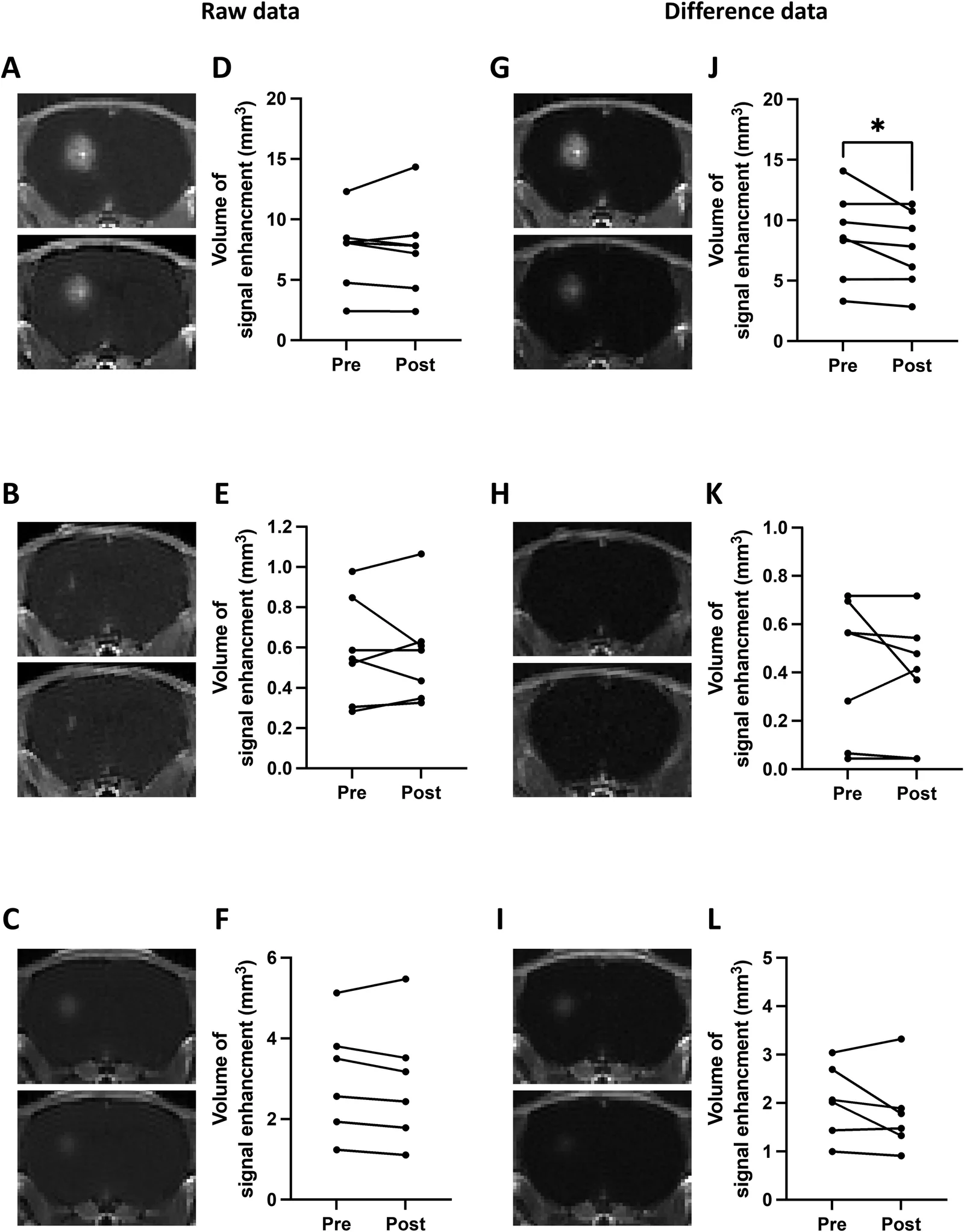
(A-C) Raw post-gadolinium T1-weighted MR images acquired both pre- and post-saline administration in mice bearing MDA231Br-GFP (A), SEBTA (B), or H1_DL2 (C) macrometastases in the left striatum. (D-F) Graphs showing paired analyses from the raw MRI data for pre- and post-saline treatment for MDA231Br-GFP (D), SEBTA (E), or H1_DL2 (F) tumor groups. (G-I) Difference post-gadolinium T1-weighted MR images acquired both pre- and post-saline administration in mice bearing MDA231Br-GFP (G), SEBTA (H), or H1_DL2 (I) macrometastases in the left striatum. (J-L) Graphs showing paired analyses from the difference MRI data for pre- and post-saline treatment for MDA231Br-GFP (J), SEBTA (K), or H1_DL2 (L) tumor groups. Wilcoxon matched pairs test *P < .05; MDA231Br-GFP *n* = 7, SEBTA *n* = 7, H1_DL2 *n* = 6. MRI, magnetic resonance imaging.

In contrast to the control animals, a significant increase in the volume of contrast enhancement in mutTNF-treated animals was apparent in the MDA231Br-GFP model, in both the raw data and difference data; mean increases of 14% (*P* < .01) and 18% (*P* < .01), respectively (Figure 2A). Similarly, the volume of contrast enhancement in the SEBTA model showed a significant increase post-treatment compared to pretreatment in the mutTNF-treated group for both analyses; mean increases of 29% (*P* < .05) and 39% (*P* < .01), respectively (Figure 2B). In contrast, in the H1_DL2 group, no significant difference was found between volumes of enhancement measured pre- and post-mutTNF treatment from the raw post-Gd-DTPA data, although a small but significant increase in the volume of enhancement became evident in the difference data, with a mean increase of 12% (*P* < 0.05; Figure 2C).

**Figure 2.**
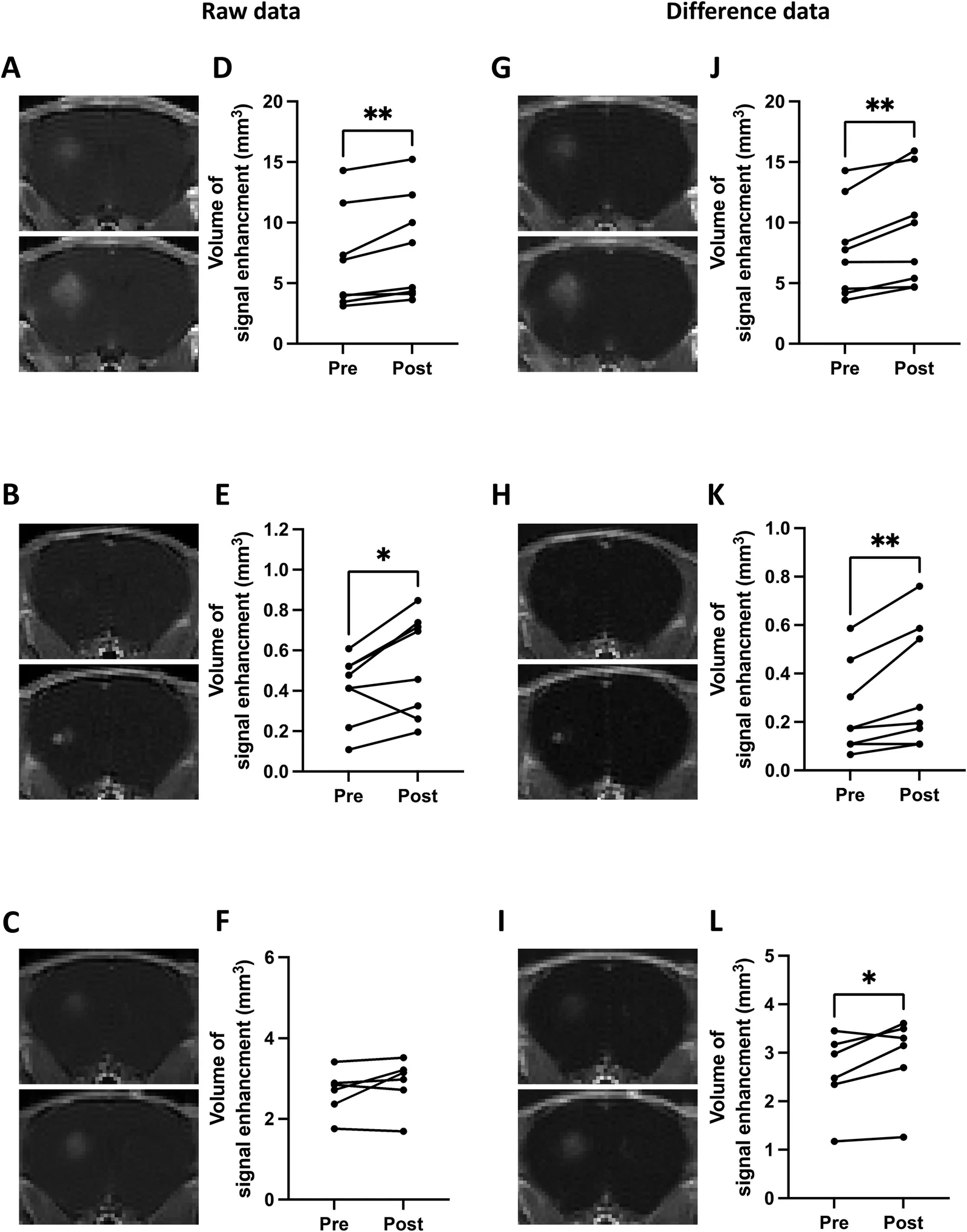
(A-C) Raw post-gadolinium T1-weighted MR images acquired both pre- and post-mutTNF administration in mice bearing MDA231Br-GFP (A), SEBTA (B), or H1_DL2 (C) macrometastases in the left striatum. (D-F) Graphs showing paired analyses from the raw MRI data for pre- and post-mutTNF treatment for MDA231Br-GFP (D), SEBTA (E), or H1_DL2 (F) tumor groups. (G-I) Difference post-gadolinium T1-weighted MR images acquired both pre- and post-mutTNF administration in mice bearing MDA231Br-GFP (G), SEBTA (H), or H1_DL2 (I) macrometastases in the left striatum. (J-L) Graphs showing paired analyses from the difference MRI data for pre- and post-mutTNF treatment for MDA231Br-GFP (J), SEBTA (K), or H1_DL2 (L) tumor groups. Wilcoxon matched pairs test *P < 0.05, **P < 0.01; MDA231Br-GFP *n* = 8, SEBTA *n* = 8, H1_DL2 *n* = 6. MRI, magnetic resonance imaging; mutTNF, TNF mutein.

### Tumor Morphology

Notably, the 3 tumor types showed marked differences in morphology and, in particular, in the degree of infiltration when assessed histologically. The MDA231Br-GFP *macro*metastases showed the most invasive profile of the 3 models, with very little solid central tumor and multiple pockets of smaller metastatic foci indicating outward growth and infiltration away from the original injection site (Figure 3A). The SEBTA *macro*metastases tended to have more of a core tumor region than the MDA231Br-GFP model, but with highly infiltrative margins (Figure 3B). In contrast to the other 2 models, the majority of the H1_DL2 *macro*metastases were very well circumscribed, particularly in the central regions, and showed relatively little infiltration away from the tumor core cross-sectionally (Figure 3C). Assessment of tumor size indicated no significant differences between treatment groups for any of the tumor models (Figures 3D-F). However, comparison between tumor models showed a range of tumor sizes, whereby MDA231Br-GFP > H1_DL2 > SEBTA (Figure 3D-F), reflecting both the different growth patterns and relative growth rates of the 3 cell types.

**Figure 3.**
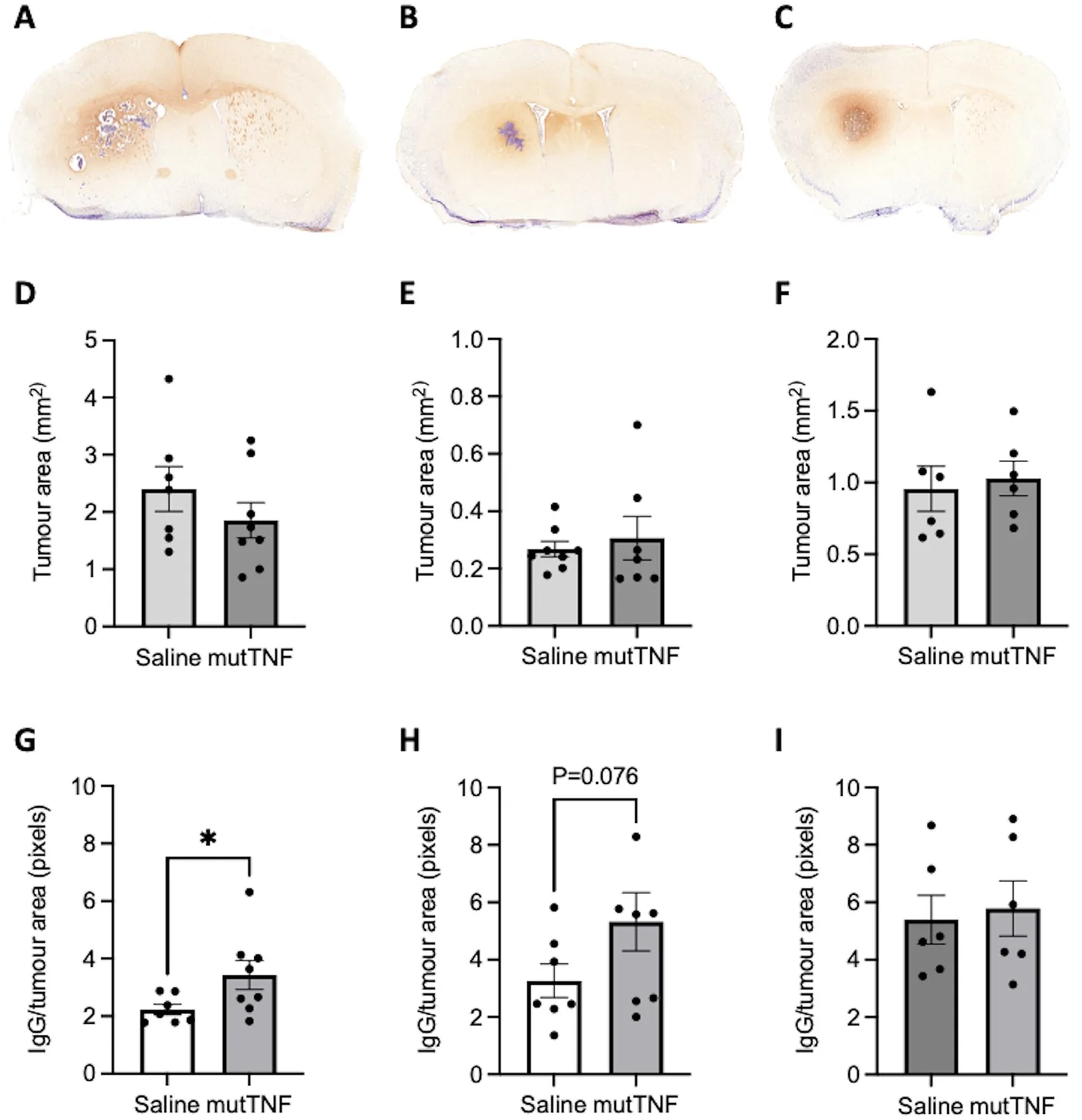
(A-C) Immunohistochemical images showing differences in morphology of the MDA231Br-GFP (A), SEBTA (B), and H1_DL2 (C) macrometastases in the left striatum of each mouse. IgG is stained brown, with a cresyl violet counterstain highlighting the tumor morphology. (D-F) Graphs showing average tumor area in the MDA231Br-GFP (D), SEBTA (E), and H1_DL2 (F) models. Welch’s *t* test showed no significant differences between treatment groups in each model. (G-I) Graphs showing average area of IgG staining normalized to tumor area at macrometastatic sites in the MDA231Br-GFP (G), SEBTA (H), and H1_DL2 (I) for saline and mutTNF-treated groups. Data are shown as mean ± SEM, together with individual points for each animal. MDA231Br-GFP saline *n* = 7, mutTNF *n* = 8; SEBTA saline *n* = 7, mutTNF *n* = 8; H1_DL2 saline *n* = 6, mutTNF *n* = 6. Mann-Whitney *U*-test, *P < 0.05. mutTNF, TNF mutein.

### Assessment of BBB Breakdown by IgG Immunohistochemistry

Differences in BBB permeabilization between mutTNF and control groups post-treatment were further determined by immunostaining for endogenous serum IgG, which is normally excluded from the brain by an intact BBB. In accord with the MRI data, a significant increase in the area of IgG extravasation detected immunohistochemically was evident in the mutTNF-treated animals compared to saline-treated animals in the MDA231Br-GFP group (mean increase = 54%, *P* < 0.05; Figure 3G). Although not significant, a strong trend toward an increase in the area of IgG extravasation was also evident in the mutTNF-treated animals in the SEBTA group (mean increase = 63%, *P* = 0.076; Figure 3H). In contrast, no difference in the area of IgG extravasation was evident between saline and mutTNF-treated groups for the H1_DL2 tumor group (mean increase = 7%; Figure 3I).

### Immunohistochemical Assessment of TNFR1 Expression

Qualitative assessment showed clear upregulation of vascular TNFR1 in all 3 models, but with noticeably different patterns of expression. In the MDA231Br-GFP model, TNFR1 expression was evident on vessels within each pocket of tumor cells and, particularly, at the foci margins (Figure 4A). However, TNFR1-positive vessels were also observed in normal-appearing brain tissue both between the foci and extending away from the edges of the tumor-bearing region (Figure 4A(ii) and (iii)). Similarly, in the SEBTA model, TNFR1 expression was clearly evident on vessels within the main tumor core and most notably at the margins, which were often well demarcated by the TNFR1 staining (Figure 4B). TNFR1 staining was also found around the edges of smaller tumor pockets breaking away from the main tumor mass, but few TNFR1-positive vessels were seen beyond the margins of the tumor (Figure 4B(ii) and (iii)). Vascular TNFR1 staining in the H1_DL2 model was altogether sparser than in the other models (Figure 4C), although strong tumor cell staining may, in part, have masked the TNFR1 staining (Figure 4C(ii)). Nevertheless, TNFR1-positive vessels were seen throughout the tumor area, again with slightly greater frequency toward the margins (Figure 4C(iii)). Almost no TNFR1-positive vessels were seen outside the tumor margins, although they were occasionally evident in the overlying cortex.

**Figure 4.**
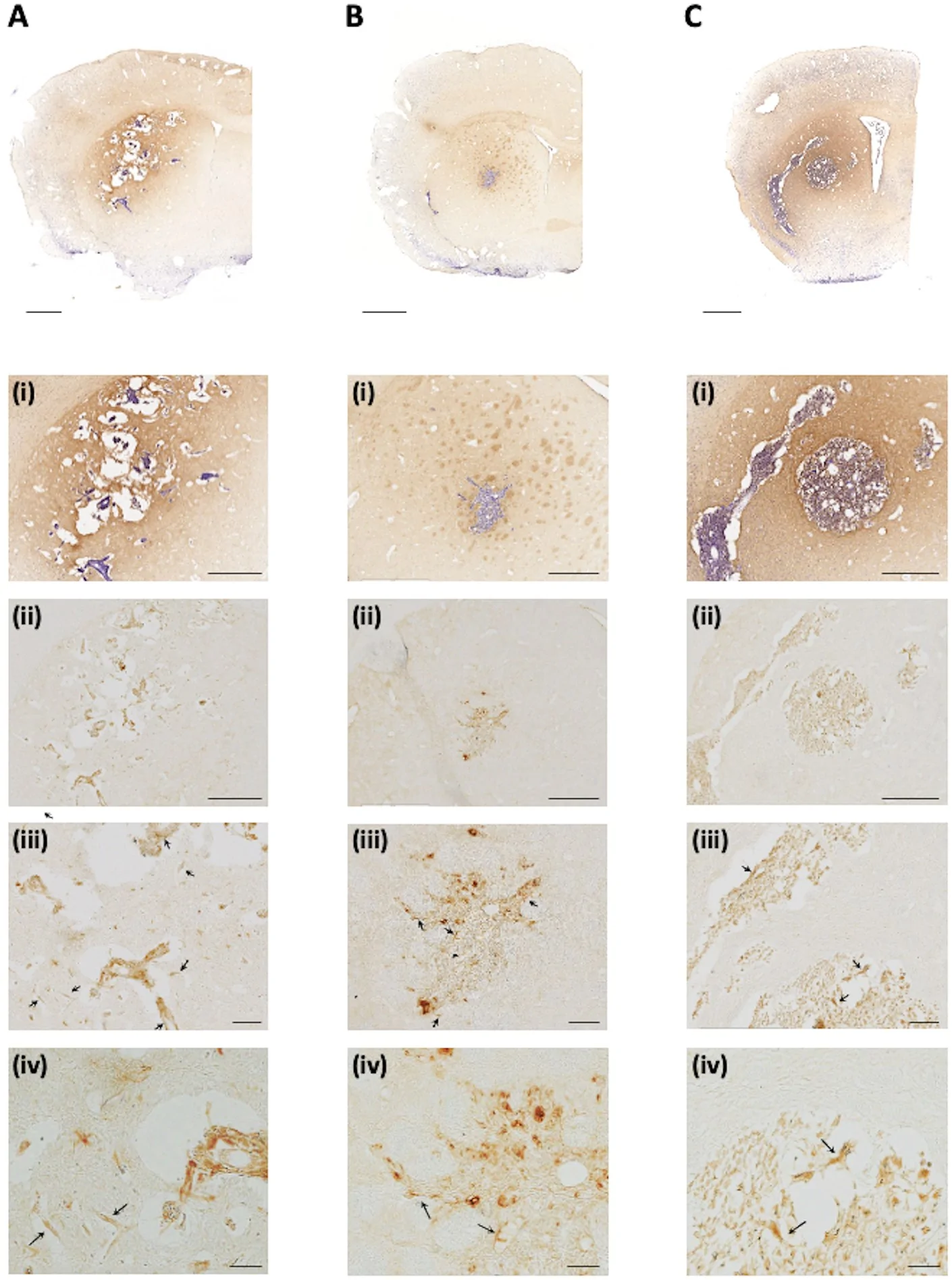
Immunohistochemical images showing differences in TNFR1 expression in the MDA231Br-GFP (A), SEBTA (B), and H1_DL2 (C) macrometastases in the left striatum of each mouse; scale bars = 1 mm. For each model (i) shows 4× magnification of tumor foci with cresyl violet counterstain and (ii) shows 4× magnification of TNFR1 staining (brown) from the adjacent section; scale bars = 500 µm. For each model, (iii) and (iv) show 10× and 20× magnification of TNFR1 staining (brown), respectively; scale bars = 100 µm and 50 µm, respectively. Arrows indicate TNFR1 positive vessels. Note in the MDA231Br-GFP model, these were frequently found outside the obvious tumor margins.

## Discussion

Prognosis for patients with brain metastases remains poor, and the BBB presents a major obstacle to effective treatment. Importantly, the BBB remains impermeant in the margins of *macro*metastatic tumors, resulting in poor delineation of tumor extent for surgical or radiotherapy treatment planning and excluding systemic therapies from these areas. As a result, relapse is common in highly proliferative, invasive margins that escape primary treatment. In this study, we have used a novel TNFR1-selective human TNF mutein (mutTNF), which we have previously shown induces de novo permeabilization of the BBB at *micro*metastatic sites in mouse brain.^11^ We now show that the same mutein enhances permeabilization of the BBB in more established *macro*metastases, with the potential to improve both delineation of tumor extent and treatment of tumor margins.

Three different models of brain *macro*metastasis were studied, from primary breast, lung, and melanoma cancers, which showed varying degrees of infiltration at the margins. The MDA231Br-GFP breast cancer model exhibited the most invasive profile of the 3 tumor types, with multiple tumor foci and little central core, and a significant increase in the extent of permeabilization following mutTNF treatment was found in both the MRI and IHC data. The SEBTA lung cancer model showed an intermediate degree of infiltration, with notably invasive margins around a central core tumor area, and in this model, a significant increase in the extent of permeabilization following mutTNF treatment was found in the MRI data, with a strong trend toward the same in the IHC data, although this did not quite reach statistical significance. By comparison, the H1_DL2 melanoma model showed the least infiltration at the margins, with a well-circumscribed tumor border, and in this model, the extent of permeabilization following mutTNF treatment only just reached significance in the MRI difference data. These findings are in accord with previous reports of the highly invasive profile of the MDA-MB-231 cell line,^20^ from which the MDA231Br-GFP cell line is derived, and our previous studies have shown a significant upregulation of both the proliferation marker Ki67 and the stemness markers SOX2 and nestin at the margins of MDA231Br-GFP brain metastases, indicating the invasive profile of these tumors in vivo.^18^ We have also shown increasing inflammatory activation in peri-tumoural vessels with tumor growth in the MDA231Br-GFP model, but not in either the SEBTA or H1_DL2 models.^21^ These findings further suggest that whilst the MDA231Br-GFP shows progressive invasion, as indicated by peri-tumoral inflammation, the SEBTA and H1_DL2 show less marginal invasiveness and, hence, lower and stable levels of peri-tumoral inflammation.

The difference in outcome between MRI and immunohistochemical analyses in the H1_DL2 and SEBTA models likely reflects, in part, the morphology of these tumors and, in part, the sensitivities of the different analysis methods. For the H1_DL2 tumors, in particular, the additional enhancement found in the difference MR images arose at the anterior and posterior ends of the tumors, with relatively little change in volume in the central section of the tumors. Consequently, owing to the method of analysis employed for the IgG IHC, in which measurements were made across 3 sections spanning the central region of the tumor, any mutTNF-induced permeabilization at the anterior and posterior edges of the tumors would not have been captured. Qualitative examination of the IgG staining showed corresponding increases in the spread of IgG staining both anterior and posterior to the tumor margins in a number of animals, in accord with the MRI data (Figure S2A-C). Moreover, further histological examination revealed that toward the anterior and posterior edges of these tumors, a more invasive profile was sometimes evident, with additional isolated tumor foci observed in several animals (Figure S2D-F).

At the same time, as all immunohistochemical data were, by default, only available post-treatment, comparisons between groups (control vs mutTNF) are likely to be less sensitive to small differences than the paired, within-animal (post- vs pre- treatment), comparisons that are possible with the MRI data. Consequently, the MRI analyses were consistently more sensitive to the effects of mutTNF in permeabilizing the metastases. The lack of significance in the raw MRI data for the H1_DL2 model is likely explained by the fact that the raw data are inherently noisier than the difference images and, consequently, the small additional areas of contrast enhancement would be less detectable.

Broadly, patterns of TNFR1 expression were in line with the changes in mutTNF-induced permeabilization. All tumors showed upregulation of vascular TNFR1, but the extent and pattern varied between models. In the MDA231BR-GFP model, TNFR1 upregulation was very evident on vessels both within and around the tumor foci, as well as spreading beyond the obvious tumor margins into normal-appearing brain tissue, the latter likely reflecting the presence of single or small clusters of tumor cells. Both the SEBTA and H1_DL2 models showed clear TNFR1 expression within the main tumor mass, and in the SEBTA model, in particular, this staining often resulted in clear definition of both margins and small foci protruding from the main tumor core. However, in both models, TNFR1 expression on vessels outside the visible tumor area was scarce. These findings support the notion that the more invasive the tumor type (such as the MDA231Br-GFP model), the greater the expression of TNFR1, particularly in marginal areas and beyond where there may be isolated tumor cells or small tumor cell clusters. Thus, as the invasiveness of the metastases increases, their sensitivity to the permeabilizing effects of mutTNF is greater. Taken together, the above findings suggest that the use of mutTNF could significantly improve both detection and treatment of tumor margins and invasive foci beyond the apparent tumor boundary. Even in tumors with a less invasive phenotype, given the propensity toward marginal TNFR1 upregulation, it is likely that mutTNF would still improve detection of these tumor boundaries.

Several other approaches to circumventing an intact BBB have been reported previously, but all suffer limitations, as discussed in detail previously.^11^ We have shown that the mutTNF used here can induce permeabilization of the BBB across a size range spanning from 0.5 kDa (HRP) to 150 kDa (IgG), which would encompass the majority of, if not all, current therapeutics in terms of size. This effect is particularly pertinent for monoclonal antibody-based therapies such as trastuzumab (145.5 kDa), pertuzumab (137.9 kDa), and durvalumab (149kDa), which have shown considerable promise in metastatic cancer but are unable to cross the intact BBB.^22^ In accord with this notion, we have also demonstrated that co-administration of trastuzumab with mutTNF significantly enhances delivery of this antibody to micrometastatic sites in the brain.^11^ Thus, combination of mutTNF with systemic therapeutics may significantly improve treatment of larger brain metastases that are currently considered incurable by enhancing the treatment of the proliferative and invasive margins.

In conclusion, the findings of this study suggest that where *macro*metastases in the brain present with an invasive profile, the tumor margins are highly sensitive to the permeabilizing effects of mutTNF. These data suggest that mutTNF has the potential not only to permeabilize *micro*metastatic tumors in the brain, as demonstrated previously,^10^^,^^11^ but also to enhance both delineation of *macro*metastatic tumor margins for treatment planning and delivery of systemic therapies to those tumor margins. Even in the case of a less-invasive phenotype, mutTNF administration may further improve detection of tumor boundaries owing to TNFR1 upregulation at tumor margins. Our previous work demonstrating a penumbra of vascular activation around human brain metastases^18^ including TNFR1 upregulation^10^ support the clinical applicability of the current findings, and together these provide further preclinical and clinical support for translation of mutTNF to the clinic.

## Supplementary Material

vdag239_Supplementary_Data

## Data Availability

Data not already included in the manuscript or supplementary materials may be obtained from the corresponding author.

## References

[1] Valiente M , Ahluwalia MS, Boire A, et al The evolving landscape of brain metastasis. Trends Cancer. 2018;4:176-196. 10.1016/j.trecan.2018.01.003 PMC6602095 29506669

[2] Eichler AF , Chung E, Kodack DP, Loeffler JS, Fukumura D, Jain RK. The biology of brain metastases-translation to new therapies. Nat Rev Clin Oncol. 2011;8:344-356. 10.1038/nrclinonc.2011.58 PMC3259742 21487419

[3] Hersh DS , Wadajkar AS, Roberts N, et al Evolving drug delivery strategies to overcome the blood brain barrier. Curr Pharm Des. 2016;22:1177-1193. 10.2174/1381612822666151221150733 PMC4900538 26685681

[4] Caraglia M , Addeo R, Costanzo R, et al Phase II study of temozolomide plus pegylated liposomal doxorubicin in the treatment of brain metastases from solid tumours. Cancer Chemother Pharmacol. 2006;57:34-39. 10.1007/s00280-005-0001-z 16010592

[5] Cote J , Bovenzi V, Savard M, et al Induction of selective blood-tumor barrier permeability and macromolecular transport by a biostable kinin B1 receptor agonist in a glioma rat model. PLoS One. 2012;7:e37485. 10.1371/journal.pone.0037485 PMC3357387 22629405

[6] Cote J , Savard M, Neugebauer W, Fortin D, Lepage M, Gobeil F. Dual kinin B1 and B2 receptor activation provides enhanced blood-brain barrier permeability and anticancer drug delivery into brain tumors. Cancer Biol Ther. 2013;14:806-811. 10.4161/cbt.25327 PMC3909549 23792591

[7] Guillaume DJ , Doolittle ND, Gahramanov S, Hedrick NA, Delashaw JB, Neuwelt EA. Intra-arterial chemotherapy with osmotic blood-brain barrier disruption for aggressive oligodendroglial tumors: results of a phase I study. Neurosurgery. 2010;66:48-58; discussion 58. 10.1227/01 PMC2806091 20023537

[8] Mainprize T , Lipsman N, Huang Y, et al Blood-brain barrier opening in primary brain tumors with non-invasive MR-guided focused ultrasound: a clinical safety and feasibility study. Sci Rep. 2019;9:321. 10.1038/s41598-018-36340-0 PMC6344541 30674905

[9] Prados MD , Schold SC Jr, Fine HA, et al A randomized, double-blind, placebo-controlled, phase 2 study of RMP-7 in combination with carboplatin administered intravenously for the treatment of recurrent malignant glioma. Neuro Oncol. 2003;5:96-103. 10.1093/neuonc/5.2.96 PMC1920676 12672281

[10] Connell JJ , Chatain G, Cornelissen B, et al Selective permeabilization of the blood-brain barrier at sites of metastasis. J Natl Cancer Inst. 2013;105:1634-1643. 10.1093/jnci/djt276 PMC3818170 24108809

[11] Munoz Pinto MF , Campbell SJ, Simoglou Karali C, et al Selective blood-brain barrier permeabilization of brain metastases by a type 1 receptor-selective tumor necrosis factor mutein. Neuro Oncol. 2022;24:52-63. 10.1093/neuonc/noab177 PMC8730757 34297105

[12] Crouzen JA , Petoukhova AL, Hakstege M, et al Patterns of recurrence after postoperative stereotactic radiotherapy for brain metastases. *Cancers*. 2025;17: 1557. 10.3390/cancers17091557 PMC12071874 40361483

[13] Patel AJ , Suki D, Hatiboglu MA, et al Factors influencing the risk of local recurrence after resection of a single brain metastasis. J Neurosurg. 2010;113:181-189. 10.3171/2009.11.JNS09659 20035574

[14] Stockham AL , Suh JH, Chao ST, Barnett GH. Management of recurrent brain metastasis after radiosurgery. Prog Neurol Surg. 2012;25:273-286. 10.1159/000331200 22236688

[15] Berghoff AS , Rajky O, Winkler F, et al Invasion patterns in brain metastases of solid cancers. Neuro Oncol. 2013;15:1664-1672. 10.1093/neuonc/not112 PMC3829586 24084410

[16] Siam L , Bleckmann A, Chaung HN, et al The metastatic infiltration at the metastasis/brain parenchyma-interface is very heterogeneous and has a significant impact on survival in a prospective study. Oncotarget. 2015;6:29254-29267. 10.18632/oncotarget.4201 PMC4745724 26299612

[17] Zakaria R , Das K, Radon M, et al Diffusion-weighted MRI characteristics of the cerebral metastasis to brain boundary predicts patient outcomes. BMC Med Imaging. 2014;14:26. 10.1186/1471-2342-14-26 PMC4126355 25086595

[18] Cheng VWT , de Pennington N, Zakaria R, et al VCAM-1-targeted MRI improves detection of the tumor-brain interface. Clin Cancer Res. 2022;28:2385-2396. 10.1158/1078-0432.CCR-21-4011 PMC9662863 35312755

[19] Soto MS , O’Brien ER, Andreou K, et al Disruption of tumour-host communication by downregulation of LFA-1 reduces COX-2 and e-NOS expression and inhibits brain metastasis growth. Oncotarget. 2016;7:52375-52391. 10.18632/oncotarget.10737 PMC5239559 27447568

[20] Amaro A , Angelini G, Mirisola V, et al A highly invasive subpopulation of MDA-MB-231 breast cancer cells shows accelerated growth, differential chemoresistance, features of apocrine tumors and reduced tumorigenicity in vivo. *Oncotarget*. 2016;7:68803-68820. 10.18632/oncotarget.11931 PMC5356591 27626697

[21] Zarghami N , Soto MS, Perez-Balderas F, et al A novel molecular magnetic resonance imaging agent targeting activated leukocyte cell adhesion molecule as demonstrated in mouse brain metastasis models. J Cereb Blood Flow Metab. 2021;41:1592-1607. 10.1177/0271678X20968943 PMC8217895 33153376

[22] Guglielmi G , Zamagni C, Del Re M, Danesi R, Fogli S. Targeting HER2 in breast cancer with brain metastases: a pharmacological point of view with special focus on the permeability of blood-brain barrier to targeted treatments. Eur J Pharmacol. 2024;985:177076. 10.1016/j.ejphar.2024.177076 39486766

