# Supplementary material for: Permeabilization of tumor margins in brain macrometastases using a type-1 receptor selective tumor necrosis factor mutein
Source: Neurooncol Adv. 2026 Sep 8;8(1):vdag239. doi: 10.1093/noajnl/vdag239 (PMC13625016; doi:10.1093/noajnl/vdag239)
Supplement: Supplementary file 1 [file vdag239_supplementary_data.zip › Johanssen et al Supplementary Figure Legends.docx]

**Supplementary Figures:**

**Figure S1: (A-F)** Raw post-gadolinium T1-weighted **(i)** and difference **(ii)** MR images acquired prior to either saline **(A-C)** or mutTNF **(D-F)** treatment in mice bearing MDA231Br-GFP, SEBTA or H1_DL2 macrometastases in the left striatum. **(G-I)** Bar graphs showing comparison of contrast enhancement volumes pre-treatment from the raw **(i)** and difference **(ii)** MRI data for the MDA231Br-GFP **(G)**, SEBTA **(H)** and H1_DL2 **(I)** tumour groups. Mann Whitney u test showed no significant differences pre-treatment between treatment groups for each model; MDA231Br-GFP saline n=7, mutTNF n=8; SEBTA saline n=7, mutTNF n=8; H1_DL2 saline n=6, mutTNF n=6.

**Figure S2:** Immunohistochemical images showing spread of IgG staining (brown) at anterior edge **(A, D)**, middle **(B, E)** and posterior edge **(C, F)** of H1_DL2 tumour, together with presence of multiple tumour foci at edges compared to the central region. Scale bars (A-C) = 1mm, (D-F) = 500mm.
